# Architecture of transcriptional regulatory circuits is knitted over the topology of bio-molecular interaction networks

**DOI:** 10.1186/1752-0509-2-17

**Published:** 2008-02-08

**Authors:** Ana Paula Oliveira, Kiran Raosaheb Patil, Jens Nielsen

**Affiliations:** 1 Center for Microbial Biotechnology, Department of Systems Biology, Technical University of Denmark, Building 223, DK-2800 Kgs. Lyngby, Denmark; 2 Current address: Department of Chemical and Biological Engineering, Chalmers University of Technology, SE-412 96 Gothenburg, Sweden

## Abstract

**Background:**

Uncovering the operating principles underlying cellular processes by using 'omics' data is often a difficult task due to the high-dimensionality of the solution space that spans all interactions among the bio-molecules under consideration. A rational way to overcome this problem is to use the topology of bio-molecular interaction networks in order to constrain the solution space. Such approaches systematically integrate the existing biological knowledge with the 'omics' data.

**Results:**

Here we introduce a hypothesis-driven method that integrates bio-molecular network topology with transcriptome data, thereby allowing the identification of key biological features (Reporter Features) around which transcriptional changes are significantly concentrated. We have combined transcriptome data with different biological networks in order to identify Reporter Gene Ontologies, Reporter Transcription Factors, Reporter Proteins and Reporter Complexes, and use this to decipher the logic of regulatory circuits playing a key role in yeast glucose repression and human diabetes.

**Conclusion:**

Reporter Features offer the opportunity to identify regulatory hot-spots in bio-molecular interaction networks that are significantly affected between or across conditions. Results of the Reporter Feature analysis not only provide a snapshot of the transcriptional regulatory program but also are biologically easy to interpret and provide a powerful way to generate new hypotheses. Our Reporter Features analyses of yeast glucose repression and human diabetes data brings hints towards the understanding of the principles of transcriptional regulation controlling these two important and potentially closely related systems.

## Background

High-throughput analytical techniques for genome-wide quantification and mapping of cellular components have brought new promises and challenges to modern biology [1-3]. One of the major challenges resides on how to analyze and extract knowledge from the vast amounts of 'omics' data being generated. Many methods have been proposed to help revealing cellular transcriptional regulatory programs by using transcriptome data, which is the most common and, so far, the only truly genome-wide type of quantitative 'omics'. Analysis of transcriptome data typically starts by filtering for genes that change their expression levels significantly, followed by grouping of these genes based on similar behavior under the studied conditions. Moreover, such analysis methods often assume that there may be an all-to-all interaction among the studied genes. Although this assumption may help to reveal new potential biological relationships, it also leads to the identification of several false positives. Such an open-end analysis with very high dimensional search space often shadows the biological logic behind the observed transcriptional changes and thus it limits the understanding of the underlying design principles of the biological system.

The dimensionality of the data analysis problem can be considerably reduced if biological information (e.g., physical and/or functional interactions between bio-molecules) is used in order to constrain the solution space (i.e., the number of possible regulatory hypotheses explaining the observed 'omics' data), hence enhancing the possibility of uncovering the biological dimensions of the data [4,5]. Therefore, integration of biological network topology with transcriptome data offers an opportunity to effectively perform modular analysis of cellular transcriptional responses. Examples of such genome-scale bio-molecular interaction information that can be readily found for several organisms include protein functional annotation, protein-protein interactions, protein-DNA interactions, protein complexes and reconstructed metabolic networks.

## Hypothesis

We report here a hypothesis-driven method, called Reporter Features algorithm, to integrate 'omics' data with the topology of biological interaction networks, and demonstrate that this method can elucidate the basic principles of regulation in these networks. We hypothesize that the topology of biological interactions itself guides (and constrains) the regulatory response of the network following a perturbation in the system. The simplest form of regulatory principle stemming from this hypothesis is that a perturbation (or a response to a perturbation) may trigger a regulatory response beginning at the first neighbors of the affected node(s), as illustrated in our earlier analysis of metabolic networks [5]. Consequently, this hypothesis can be used to understand the modes of action of cellular regulatory mechanisms by identifying key regulatory nodes around which the response is significantly concentrated. Our Reporter Features algorithm identifies groups of neighbor genes (i.e., genes associated with a certain feature) that are significantly and collectively co-regulated compared to the background, and this concept can be easily extended to any *n*^*th *^degree neighbors. Notably, the Reporter Features algorithm does not require *a priori *decision on what changes are or are not significant at the level of each node (e.g., the transcript of a gene). Here, we present evidence that support our hypothesis and further illustrate the applied power of Reporter Features in identifying responsive biological functional modules, by determining Reporter Gene Ontologies, Reporter Transcription Factors, Reporter Proteins and Reporter Complexes for yeast and human transcriptional datasets. Moreover, we introduce different scoring systems to assign statistical significance to the features under investigation, each yielding different interpretations of what the feature significance is.

## Algorithm

**Figure 1:**
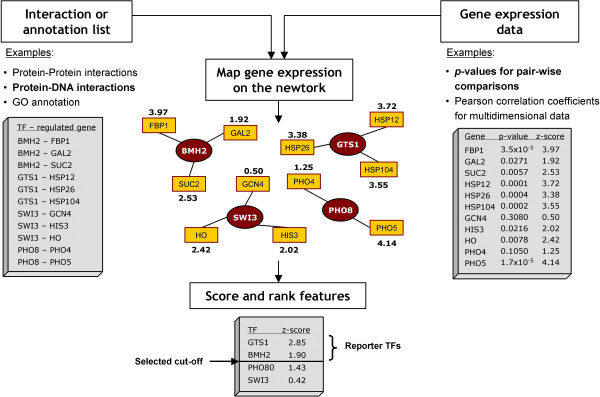
**Illustration of the Reporter Features algorithm**. The algorithm takes as input gene expression data (significance of change for pair-wise comparisons or correlation for multi-dimensional datasets) and the topology of bio-molecular interaction networks (either physical or functional interactions) represented as a graph. The bio-molecular network should be of the type "*feature j *- *gene i*", where *feature j *is any biological feature of interest (e.g., a GO term, a transcription factor, a protein or a metabolite) associated with the *gene i*. Gene expression in the form of *z*-score is then mapped onto the "gene nodes" of the graph. Finally, the score of each feature can be calculated based on the score of its neighbors "gene nodes" (see Methods). Reporter Features are those features with a *z*-score above a selected cut-off. The example in the grey boxes was selected from the Reporter TFs for the Δ*grr1 *dataset.

The Reporter Features algorithm is a generalization and extension of the Reporter Metabolites algorithm that we have previously reported [5]. Figure 1 depicts the principles of the proposed algorithm, which is described in detail in the following.

### (i) Representation of interaction/annotation lists as bipartite graphs

**Figure 2:**
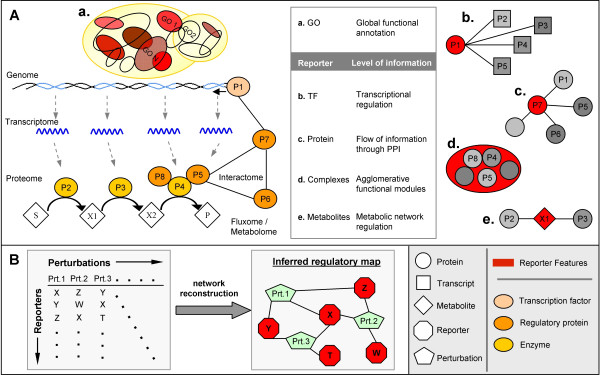
**The multi-level nature of Reporter Features**. A) Different Reporter Features can be calculated using different network representations of biological information, which will capture different but complementary aspects of the functionality of cellular machinery. Reporter GOs (marked in red tones in (a.)) are those Gene Ontology categories whose corresponding genes are most responsive to a perturbation than the background, and indicate which global functional groups within the cell are responding to the perturbation. Reporter TFs (b.), Reporter Proteins (c.), Reporter Complexes (d.) and Reporter Metabolites (e.) provide insights into more specific mechanistic aspects of the cellular response. For example, for the Reporter TFs network (b.), the transcription factor P1 is connected to all genes under its transcriptional regulation, i.e., transcripts of P2, P3, P4 an P5, this way depicting the regulatory network working through P1. B) For a series of related perturbations, Reporter Features allow the reconstruction of inferred regulatory maps that unify the perturbations under study *via *corresponding common regulatory mechanisms underlying them.

Graph-theoretical representation of biological information has brought new capabilities to the analysis of 'omics' data [4-6]. For the present method, it is of particular interest to note that both bio-molecular interaction networks and annotation lists can be represented as bipartite graphs whenever the correspondence "feature *j *- gene *i*" can be established (or, more generally, "feature *j *- molecule *i*"). In bipartite undirected graphs, both features and genes are represented as nodes, and interactions between them are represented as edges. Therefore, a gene will be connected to all features for which the correspondence "feature *j *- gene *i*" exists (Figure 1). In the case of Reporter Metabolites, this association is "metabolite *j *- gene *i*" [5], meaning that all metabolites involved in a reaction catalyzed by a certain gene product were connected to the corresponding gene. To illustrate the generalization of the Reporter Feature algorithm, we use information derived from gene ontology annotation databases ("gene ontology *j *- gene *i*"), transcription factor-DNA interaction networks ("transcription factor *j *- gene *i*"), protein interaction networks ("protein *j *- protein *i*") and protein complexes composition ("complex *j *- gene *i*") to determine the so called "Reporter Gene Ontologies", "Reporter Transcription Factors", "Reporter Proteins" and "Reporter Complexes", respectively (Figure 2A).

### (ii) Mapping and scoring of transcription data

Cellular molecules such as mRNA, proteins and low molecular-weight metabolites can be quantified at 'omics' level, and this information can be mapped onto the corresponding nodes in a bipartite graph representation of the selected bio-molecular interaction network. To illustrate the proposed method, we deal here with transcriptome data.

When analyzing gene expression data comparing two conditions, a pair-wise assessment for the significance of change per transcript can be determined by using, for instances, a Student's *t*-test, and calculating the corresponding *p*-value. Each *p*_*gene i *_can then be converted into a *z*-score by using the inverse normal cumulative distribution function (*cdf*^-1^). Thus, in case of uniformly distributed *p*-values (random data assumption), the resulting *z*-scores will follow a standard normal distribution.

*z*_*gene i *_= *cdf*^-1 ^(1 - *p*_*gene i*_)

### (iii) Scoring a feature

After scoring each non-feature node, we need to calculate the score of each feature *j*, z_feature j_. We propose two scoring systems, one based on the distribution of means of random groups of the same size, the other based on evaluating how the distribution of the scores of neighbor nodes compares to the distribution for all nodes.

a) Score based on distribution of means

This scoring system has been widely used, e.g. by Ideker et al (2002) and Patil and Nielsen (2005) [4,5], and in this context it is a test for the null hypothesis "genes adjacent to feature *j *display their normalized average response by chance". In particular, the score of each feature *j *is defined as the average of the scores of its neighbor nodes (genes), i.e.:

$$z_{feature\;j}=\frac{1}{N}\sum_{k=1}^{N}z_{gene\;k}$$

To evaluate the significance of each z_feature j_, this value should be corrected for the background distribution of *z *scores in the data, by subtracting the mean (*μ*_N_) and dividing by the standard deviation (*σ*_N_) of random aggregates of size *N*. We choose the number of random samples sufficiently high (10000), this value being determined by checking the sensitivity of the resulting background scores to the (increasing) number of random samples. We here note that, due to the Central Limit Theorem, the background distribution of the scores quickly approaches a normal distribution with the increasing *N*, where the group mean equals the sample mean (independent of *N*) and *σ*_N _equals the sample standard deviation divided by $\sqrt{N}$. The transformation of *p*-values to *z*-scores (equation 1) only helps to ensure the better normality of the background scores, even for relatively small *N *(since the distribution of individual gene *z*-scores will be approximately normal). To obtain *σ*_N _as a smooth function of *N*, we fitted the results of random sampling to the power function in *N*. This way, the score of each feature is also size-independent (Central Limit Theorem, also see [4]).

$$z_{feature\;j}^{corrected}=\frac{\left(z_{feature\;j}-\mu_{N}\right)}{\sigma_{N}}$$

Reporter Features will then correspond to the features that score higher. Since $z_{feature\;j}^{corrected}$ can be converted back into *p*-values using the normal cumulative distribution function, the desired level of significance can be set by the user to define what should be considered as 'Reporter'.

b) Score based on distribution for all nodes *versus *distribution for neighbors

Alternatively, one can perform a statistical comparison test (e.g., a *t*-test or a non-parametrical test such as u-test) to assess whether the distribution of the adjacent nodes' scores of a certain feature *j *differs from the distribution of scores for all nodes. In this case, a *p*-value can be calculated for each feature *j *based on the probability that the null hypothesis (equal distribution) is true. Reporter Features will therefore correspond to the features with lower *p*-values.

Evaluation of the two different scoring systems, and a few additional proposed scoring systems, is described under Results and Discussions, as we hereby can put the scoring systems in a biological context.

### (iv) Higher-degree Reporters

The above described scoring system leads to what we termed *first-degree *Reporters (*n *= 1). Other *n*^*th *^*degree *scoring systems can also be implemented which accounts for the response affecting farther than the immediately adjacent nodes.

Extending the scoring system previously described, the resulting *z*-score of *n*^*th *^*degree *for the feature *j *is defined as:

$$\begin{array}{c} n \end{array}z_{feature\;j}=\frac{1}{M}\sum_{k=1}^{M}z_{node\;k}\text{ , }f\text{or all }k\text{: shortest path length(}feature\;j\text{, }node^{k})\leq n,$$

where *M *is the total number of neighbors of degree equal to or less that *n*.

Similarly to the *first-degree *case, ^*n*^*z*_*feature j *_should be corrected for background:

$$\begin{array}{c} n \end{array}z_{feature\;j}^{corrected}=\frac{\left(\begin{array}{c} n \end{array}z_{feature\;j}-\;\begin{array}{c} n \end{array}\mu_{N}\right)}{\begin{array}{c} n \end{array}\sigma_{N}}$$

### (v) Use of information on up/down regulation of non-feature genes

The graphs and scores we have been considering are undirected, i.e., they do not account for directionality of neither the feature-gene interaction nor the change in the non-feature node property (e.g. up/down regulation of a transcript). However, sometimes it may be biologically relevant to consider the direction of change (up/down regulation with respect to reference condition) when determining Reporter Features. In such cases, we preprocessed the initial dataset to filter only for the desired information (i.e., including only genes that are up or down-regulated). The result is a new network that is a sub-graph of the initial graph where only up (/down) regulated genes are included. Corresponding Reporter Features can be used to compare the ranking of desired features with and without incorporation of the up/down regulation information and thereby further enrich the information about the biological role of a perturbation. This simple filtering, however, is not information-preserving. For example, in a given dataset, a TF feature may be connected to *U *up-regulated and *D *down-regulated genes. Filtering out *U *(or *D*) genes will necessarily lead to loss of information for that TF unless and until either *U *or *D *is zero. Hence the scores obtained from these sub-graphs must be analyzed only in complementation with the results from the whole graph and values of *U *and *D*.

### (vi) Inferred regulatory maps

When Reporter Features are applied to a series of related perturbations, results can be used to construct an inferred regulatory map reconnecting physical or functional interactions between the perturbed elements (Figure 2B). In this network, each perturbed element is linked to the Reporter Features calculated from the corresponding perturbation data. The resulting network is a representation of direct and/or indirect mechanisms of regulation that span the set of (related) perturbations used.

## Results and Discussion

As a proof-of-concept, we first analyzed data related to glucose repression in the yeast *S. cerevisiae*. Glucose repression refers to the capacity of the cell to sense glucose and consequently control the transcriptional response of genes involved in the utilization of alternative carbon sources. Because of its role in nutrient sensing and relevance to metabolic diseases such as diabetes [7], glucose repression serves as a model system for studying signaling and transcriptional regulation [8-10]. We applied the Reporter Feature algorithm to analyze the transcriptional response of different *S. cerevisiae *mutants with deletions in key components of glucose repression (namely Δ*grr1*, Δ*hxk2*, Δ*mig1*, Δ*mig1mig2 *and Δ*rgt1 *[9,11]). By using available physical and functional interactions in yeast – a protein interaction network, the composition of protein complexes, a transcription factor/effectors regulatory network and the Gene Ontology annotation – we determined Reporter Proteins, Reporter Complexes, Reporter Transcription Factors (TFs) and Reporter Gene Ontologies (GOs), respectively (Figure 2A), for the different gene deletion experiments. All results are available in the Additional file 1.

### Reporter GOs for yeast glucose repression mutants

Reporter GOs provide extensive functional characterization on which of the biological processes, molecular functions or cellular components are most affected at transcriptional level in response to a perturbation, without significant *a priori *knowledge. Therefore, Reporter GOs convey by themselves direct functional information on the perturbed element (Additional file 2). For the Δ*grr1 *and Δ*hxk2 *mutants, Reporter GOs overlap to a large extent, albeit in different order. For these mutants, the top-30 Reporter GOs are mostly associated with respiration, mitochondrial activities, TCA cycle and hexose transporters, which are also 'Reporter GOs for Up-Regulated Genes Only'. Hexose transporters are also 'Reporter GOs for Down-Regulated Genes Only'. Together, this indicates that the de-repression of genes related to respiration (which are usually repressed in the presence of high levels of extracellular glucose) and changes in hexose transporters utilization are the main transcriptional changes occurring in response to the *GRR1 *and *HXK2 *single deletions, and this agrees with the physiological observation that Δ*grr1 *and Δ*hxk2 *have a lower glucose uptake rate and a higher yield of biomass on substrate than the reference strain [12]. For the Δ*mig1mig2 *mutant, the top-30 Reporter GOs are also associated with respiration, mitochondrial activities and TCA cycle, supporting the knowledge that Mig1 and Mig2 are transcription factors involved in these processes when cells are growing in media containing high levels of glucose [9]. Lastly, low Reporter GO scores for the Δ*mig1 *mutant highlight that the deletion of *MIG1 *has relatively small effects, as also observed in studies on the operation of the metabolic network [13]. Interestingly, the GO category "molecular function unknown" ranked high for the Δ*mig1 *mutant, suggesting that many genes affected by the deletion of *MIG1 *still have an unidentified function.

### Reporter TFs for yeast glucose repression mutants

To further illustrate the principles of Reporter Features we reconstructed a graph depicting each known yeast transcription factor or regulatory protein connected to all genes known to be effected by these proteins, derived from YPD [14]. Using this graph we could identify Reporter TFs (or more correctly Reporter Regulators), for which the corresponding scores provide a measure of the degree of transcriptional regulation exerted. Reporter TFs highlight the regulatory pathways affected following a perturbation, and thus uncover the functional links between the perturbation and the following regulatory mechanisms invoked in the cell. For example, Reporter TFs for the Δ*grr1 *mutant include transcription factors involved in regulation of respiration (Hap2/3/4/5 complex), regulation of stress elements (Msn2/4), regulation of chromatin remodeling (Snf2, Swi1 and Hda1) and regulators of hexose transporters (Grr1 and Rgt1). These regulators are known key players in the cellular regulatory machinery affected by the deletion of *GRR1 *[11,15].

To account for the directionality of regulation, we further analyzed the Δ*grr1 *data by determining 'Reporter TFs for Up-Regulated Genes Only' and 'Reporter TFs for Down-Regulated Genes Only'. From the sub-graph of genes that are up-regulated, genes under regulation of the Hap2/3/4/5 complex are the most significantly up-regulated, suggesting that the single deletion of *GRR1 *leads to the transcriptional de-repression of respiratory genes. Other genes significantly de-repressed in this mutant are those under the effect of regulators of stress elements (Msn2/4 and Snf1), sporulation (Snf1, Ras2), nitrogen starvation (Ras2) and chromatin remodeling (Snf2, Swi1). On the other side, 'Reporter TFs for Down-Regulated Genes Only' include regulators of amino acid metabolism (Bas1, Gcn4, Ptr3), phosphate metabolism (Pho2), iron utilization and homeostasis (Fet3 and Rcs1), hexose transporters (Rgt1) and DNA metabolism (Snf2, Rfa1, Rfa3). Additionally, the deleted gene product, Grr1, also appears as a 'Reporter TF for Down-Regulated Genes Only', confirming the positive regulatory role of Grr1. Notably, Δ*grr1 *cells have been shown to have an altered cellular morphology (more elongated than the reference strain), probably due to defective bud formation [16]. The two Reporters for 'Down-Regulated Genes Only' Rfa1 and Rfa3, which are involved in DNA replication and repair, and whose null mutants show defective budding, and the Reporter TF Snf2, also involved in DNA metabolism, represent good hints for the genetic causes of the observed altered morphology of the mutant. Identified Reporter TFs also suggest that the Grr1 may be one of the connections between nitrogen starvation and invasive yeast growth, characterized by the agglomerative behavior [15]. Overall, Reporter TFs for Δ*grr1 *are in very good agreement with the functional description of Grr1 as being involved in carbon catabolite repression, glucose-dependent divalent cation transport, high-affinity glucose transport, regulation of amino acids transport and morphogenesis. Moreover, our analysis provides new insight into the genetic basis for observed morphological changes in the Δ*grr1*mutant.

**Figure 3:**
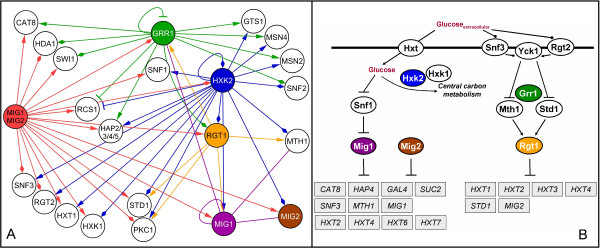
**Glucose signaling and regulatory pathway in the yeast *S. cerevisiae***. A) Inferred regulatory interaction map for glucose repression. We used genome-wide gene expression data from different mutants with deletions in key glucose repression elements (Δ*grr1*, Δ*hxk2*, Δ*mig1*, Δ*mig1mig2 *and Δ*rgt1*) [9,11] to determine Reporter TFs (*p*-value < 0.01; see Additional file 1). The graph links the deleted elements (colored nodes) to the corresponding Reporter TFs. The end arrow indicates if the protein is also a 'Reporter TF for Up-Regulated Genes only' (triangular arrow), a 'Reporter TF for Down-Regulated Gene Only (T arrow), both (diamond arrow) or none (no arrow). B) The two main pathways reported in literature for glucose sensing and signaling in yeast.

By combining the information about each deleted gene and their corresponding Reporter TFs, we constructed an inferred regulatory interaction map for glucose repression in yeast (Figure 3). The resulting map is a representation of direct and indirect signaling/regulatory cascades, and therefore it can be used as a backbone for more extensive physical interaction reconstruction. Notably, the constructed map includes most of the elements known to be involved in glucose signaling/regulatory pathways [9]. Moreover, much regulatory information is correctly captured by the map, such as the connectivity between different glucose signaling pathways and the repressing effect of Mig1 and Mig2 in genes regulated by Hap2/3/4/5 and by Cat8.

**Figure 4:**
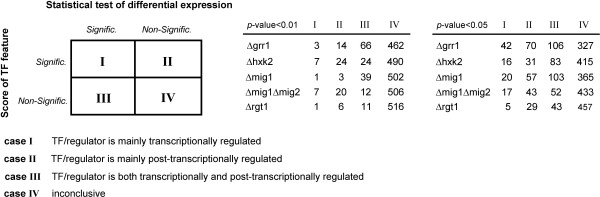
**Inferring the level of regulation of TFs from Reporter analysis**. The activity of a TF/regulator cannot be usually inferred directly from the change in gene expression, since many of these regulators are regulated post-transcriptionally. The level of regulation of a TF/regulator can be inferred based on the combined analysis of its differential gene expression and its score as a Reporter TF. Analysis of Δ*grr1*, Δ*hxk2*, Δ*mig1*, Δ*mig1Δmig2 *and Δ*rgt1 *data at two different thresholds of significance (*p*-value < 0.01 and *p*-value < 0.05) consistently shows that only few TFs are mainly transcriptional regulated (case I), while most are post-transcriptionally regulated (cases II and III).

Reporter TFs indirectly quantify TF's transcriptional regulatory activity, and this is particularly relevant since many TFs and regulators do not respond at transcriptional level *per se*, but through post-translational regulation. In particular, the level of regulation can be evaluated based on whether a regulator is a Reporter TF, and whether the same regulator has its differential expression changed significantly. This will lead to 4 different possible cases (Figure 4): (I) when the regulator is differently expressed and is also Reporter TF, indicating the regulator activity is mainly transcriptionally governed; (II) when the regulator is not differentially expressed but is a Reporter TF, suggesting that the regulator is mainly post-transcriptionally regulated; (III) when the regulator is differentially expressed but is not a Reporter TF, suggesting that the regulator is both transcriptionally and post-transcriptionally regulated; and (IV) when the regulator is neither differently expressed nor a Reporter TF, in which case no conclusion can be made regarding where the control lies. As shown in Figure 4, we found that most of the regulators in the studied examples are post-transcriptionally regulated (cases II and III), while few are only transcriptionally regulated (case I). This hints to the pitfalls of inferring the regulatory activity of a regulator based solely on its gene expression. Therefore, Reporter TFs are a valuable tool to make a better estimate of the change in TF activity following a perturbation, while providing clues whether this regulation happens at transcriptional level or downstream. Consequently, Reporter TFs provide a useful computational framework for reconstruction of regulatory circuits without *a priori *requirement of change in the transcription level of the regulators.

### Reporter Proteins for yeast glucose repression mutants

Many cellular processes involve more than one level of information processing, such as in the cases of signaling cascades, hubs of regulatory information transfer and functional associations. To probe such processes, we have determined 1^st ^and 2^nd^-degree Reporter Proteins by using the topology of the protein interaction network to identify hot-spot proteins. For the Δ*grr1 *mutant (Additional file 3), the top-10 1^st^-degree Reporter Proteins are mainly related with respiration (Atp1, Atp2, Atp6, Atp7, Atp17 and Atp18) and protein biosynthesis in the mitochondria (Mrp4 and MrpL7), which is in good agreement with the increased respiratory capacity observed for this mutant. The other two Reporter Proteins are the protein phosphatase Cdc14 and the nucleotide exchange factor Fes1. Inspection of the neighbors of Cdc14 (proteins mainly associated with three biological processes categories: organelle organization and biogenesis, cell cycle and generation of precursor metabolites and energy) suggests that Cdc14 is a key node connecting some of the most affected processes throughout the cell. On the other hand, the neighbors of Fas1 are mainly associated with stress responses and DNA repair. These transcriptional changes are probably due to the increased levels of oxygen-related DNA damage resulting from the increased level of respiration in the Δ*grr1 *mutant. Furthermore, the top-10 2^nd^-degree Reporter Proteins for the Δ*grr1 *mutant are all related with mitochondrial processes and mainly involved in the ATP synthase complex (Tim11, Atp4, Atp5, Atp6, Atp7, Atp11 Atp16, Atp17 and Atp18).

Together, 1^st ^and 2^nd^-degree Reporter Proteins again show that the deletion of *GRR1 *has a major impact on the transcription of genes related with respiration and mitochondrial protein biosynthesis, and these are co-regulated as a cluster of interacting proteins.

### Reporter Complexes for yeast glucose repression mutants

Protein complexes play a key role in the structural, orchestrated response of the cells to a perturbation and thereby represent one of the central entities in cellular modularity. We have used the MIPS database to establish a network of genes associated *via *protein complexes, including those derived from high-throughput immuno-affinity purification (followed by mass spectrometry) studies [17-19]. Integration of transcriptome data with protein-complex network yielded Reporter Complexes that identify the key protein complexes that are being transcriptionally regulated in response to specific genetic perturbations under investigation. Importantly, a high Reporter score for a particular protein complex signifies either a co-regulation of the whole complex or a very significant contribution (change in expression) from one or few genes in the complex, this way allowing the identification of both just-in-time synthesis and just-in-time assembly complexes, as postulated by Lichtenberg and colleagues [20]. We applied Reporter Complexes to analyze the transcriptional response of Δ*grr1 *and Δ*hxk2 *mutant when comparing with the reference strain [11] (Additional file 4). Similarly to Reporter GOs, Reporter Complexes for these two mutants overlap to a large extent, although in a different order, and they are mainly associated with mitochondrial ATPases and ribossomal complexes, cytochrome *c *and succinate dehydrogenase complex. Unique traits of each mutant can be inferred from distinguishable Reporter Complexes: for Δ*grr1*, Complex Number 37 from [17] (CDC14, ADK1, ATP3, ATP5, ATP7, DPM1, FUR1, GLC7, HEF3, HMS1, MCR1, PDR13, SNF4, SPE3, TPS1, VAS1, YDR453C) scores much higher that in any other mutants; for Δ*hxk2*, the mitochondrial splicing complex ranks slightly higher. Reporter Complexes complements the information obtained from Reporter Proteins, representing a more modular insight into the functional response of the cell after a perturbation.

### Reporter GOs for human diabetes data

To further illustrate the wide applicability of Reporter Features we analyzed transcriptome data from a human diabetes study [21], in which the transcriptional responses associated with type 2 diabetes mellitus were identified through quantification of mRNA levels of skeletal muscle cells from diabetic subjects (DM), insulin-resistant non-diabetic subjects (family history positive, FH^+^) and control non-diabetic subjects (family history negative, FH^-^). We applied Reporter GOs to analyze all possible pair-wise comparisons (Additional file 5). The Reporter GOs analysis was in very good agreement with the knowledge-based analysis carried out by Patti *et al *[21], while also offering additional insights. For all three comparisons, the top-10 Reporter GOs categories include the cellular components mitochondrion and ribosome, the molecular functions RNA binding and structural constituent of ribosome, and the biological processes protein biosynthesis, ubiquitin cycle, ubiquitin-dependent protein catabolism and muscle development, which are the common biological denominators marking the changes (Additional file 6). Other GO terms that were found in the top-30 Reporter GOs for the comparison 'diabetic patients vs control FH^-^' include the molecular functions DNA-directed RNA polymerase activity, hydrogen-transporting ATPase activity, cytochrome-*c *oxidase activity and NADH dehydrogenase (ubiquinone) activity, and the biological processes ATP synthesis coupled proton transport and generation of precursor metabolites and energy. Analysis of [FH^+ ^vs FH^-^] potentially isolates the changes caused by insulin-resistance. For this comparison other high-ranking GO categories includes terms related with the proteosome, ATP synthesis coupled proton transport, cytochrome-c oxidase activity, glycogen metabolism, TCA cycle and NADH dehydrogenase (ubiquinone) activity. Finally, changes uniquely associated with hyperglycemia effects can be further evaluated from the analysis of Reporter GOs for the [DM vs FH^+^] case, with most of the Reporter GO terms being similar to the Reporter categories for the previous comparisons. Notably, when comparing DM versus FH^+ ^to identify changes uniquely associated with hyperglycemia, two GO terms not reported in the original study were identified – the molecular functions enoyl-CoA hydratase activity and epoxide hydrolase activity. Gene products with these functions are involved in lipid metabolism, and were found to be up-regulated during diabetes and starvation in independent studies [22,23].

**Figure 5:**
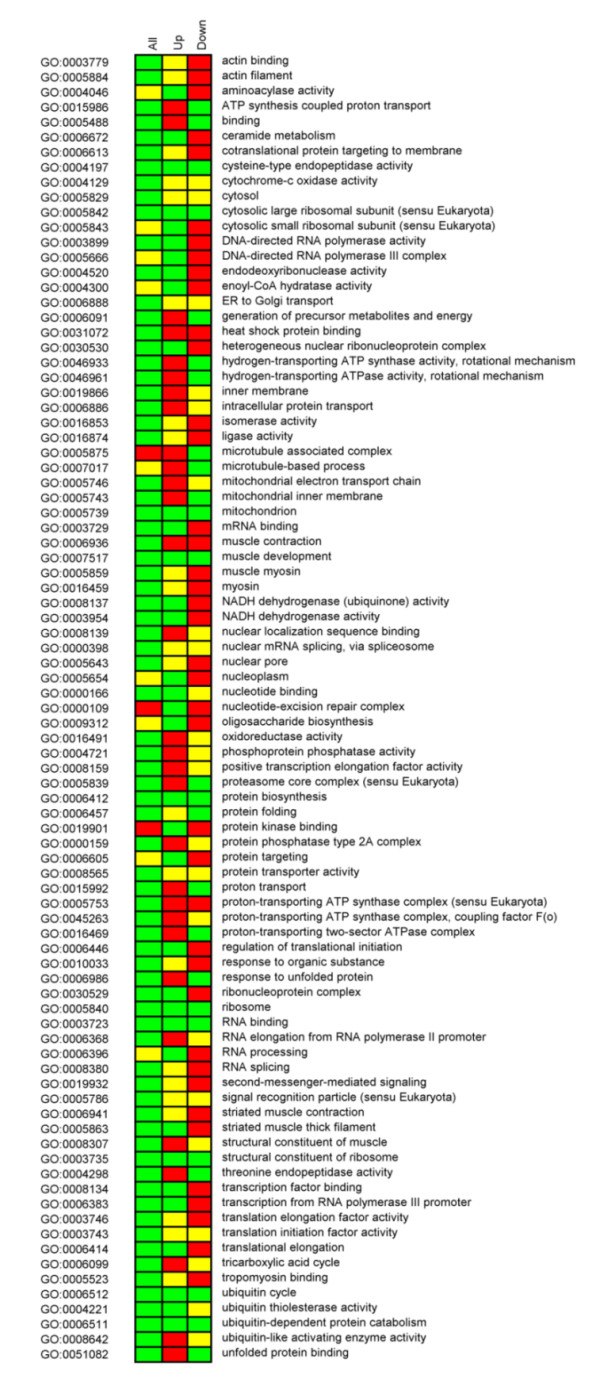
**Comparative ranking analysis**. Each column corresponds to the three human GO networks analyzed here: the complete GO network (ALL), the GO network of up-regulated nodes only (UP) and the GO network of down-regulated nodes only (DOWN). The GO terms are colored based on scores (green: *p*-value < 0.01; yellow: 0.01 <*p*-value < 0.05; red: *p*-value > 0.05), and all Reporter GO terms with a *p*-value < 0.01 in at least one of the networks are displayed. Genes belonging to the GO terms DNA-directed RNA polymerase activity, ceramide metabolism and NADH dehydrogenase (ubiquinone) activity are mostly up-regulated in the diabetic patients, while genes belonging to the GO terms generation of precursor metabolites, mitochondrial inner membrane, TCA cycle and glycolysis are mostly down-regulated in diabetic cases.

An interesting analysis is to rank GO categories by descendent scores, in both up and down-regulated sub-graphs, and evaluate if there is any dominant direction of regulation (Figure 5). This analysis revealed that the subunits of the NADH-ubiquinone oxidoreductase (part of the Complex I of the mitochondrial electron transport chain) are mostly up-regulated in insulin-resistant subjects compared to the control group. But when comparing DM and FH^+ ^subjects, diabetic patients have lower transcriptional levels of NADH-ubiquinone oxidoreductases genes, while genes encoding for cytochrome-*c*, ATP-synthesis coupled proton transporters, TCA cycle and glycolysis show an increased expression level relative to non-diatebetic FH^+ ^subjects. Although it is known that in common forms of type 2 diabetes mellitus there is a reduced activity of glycolysis, TCA cycle, *β*-oxidation, electron transport enzymes and many mitochondrial activities [21,24], Heddi and colleagues showed that, in diabetes subjects with mitochondrial DNA mutations, there is an increase in the transcript levels of many of those genes [25]. Remarkably, Reporter GOs independently point towards the same conclusions. These findings suggest that human skeletal muscle cells attempt to compensate their genomic defects by stimulating transcription of the corresponding genes.

### General applicability of Reporter Features

For the yeast *S. cerevisiae*, a model eukaryotic microorganism, there is extensive information on bio-molecular interactions and functional annotations. Thus, we used information from protein interaction databases, regulators lists and gene ontology annotation to determine Reporter Complexes, Reporter Proteins, Reporter Transcription Factors and Reporter Gene Ontologies. On the other side, most bio-molecular interactions in human cells are still poorly described, but gene ontology annotation is available for most human genes [26] as well as for many other sequenced organisms [27]. Therefore, Reporter Gene Ontology is an example of a Reporter Feature that can be applied to less well-characterized organisms, bringing valuable insights to data interpretation, as shown in the human diabetes example. Nevertheless, as more and more knowledge is added to the encyclopedia of life, better annotations and more complete networks of bio-molecular interactions will be made available for virtually all organisms, concomitantly enhancing the use of the various Reporter Features.

For determination of Reporter Gene Ontologies we have used the "simplified" version of the GO annotation as available from the GO gene-association file (which does not consider the parent-child relationship). We have also analyzed the data by using the "complete" annotation, i.e., including all parental terms (results not shown), and this analysis yielded similar results but with more repetitions of similar terms and with high-hierarchical terms coming up as Reporters more often.

### Alternative scoring systems

**Figure 6:**
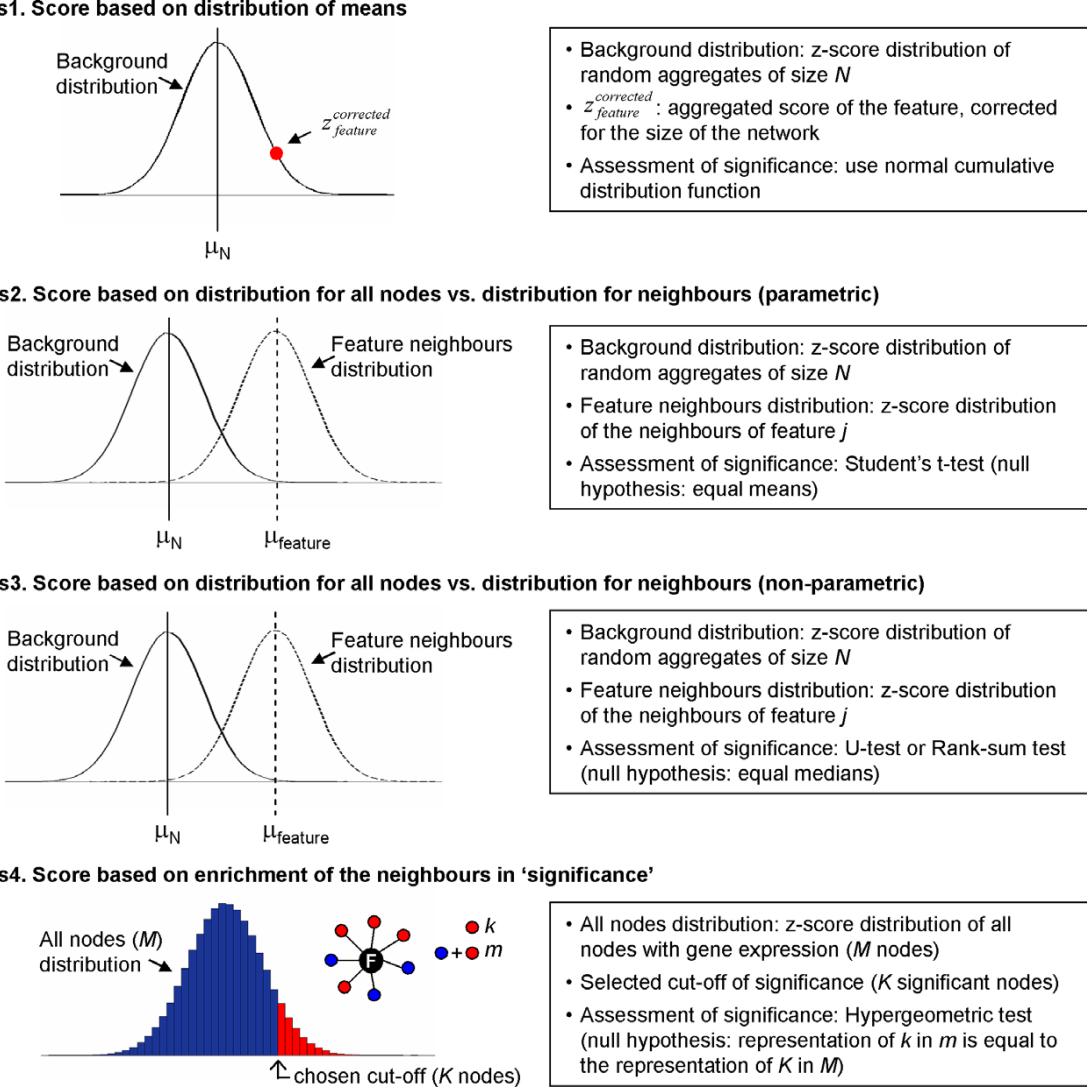
Summary of the different scoring systems suggested for Reporter analysis.

Identification of key responsive nodes in a biological interaction network relies on assigning a statistical score to each feature node. Such a score must be calculated in a biological meaningful way. Thus, we propose four different scoring systems that align with our biological hypothesis underlying the Reporter concept, which are depicted in Figure 6:

*S1*. Comparison of means (see Algorithm);

*S2*. Comparison of distribution of scores between (n^th^-degree) neighbors and background nodes, using *t*-test (see Algorithm);

*S3*. Same as *S2*, but with a non-parametric test such as rank-sum test;

*S4*. Hyper-geometric test, in which the output is the significance of having a certain number of neighbors that pass an *a priori *defined threshold *p*-value.

The results presented so far have been determined by using a scoring system *S1*, that matches closely with the definition of Reporter Features. The use of scoring systems *S2*, *S3 *and *S4 *is recommended as a complement to the analysis of Reporter Features determined by using *S1*. To illustrate the use of the different scores for interpretation of Reporter results, we present all four proposed scores for the Δ*grr1 *and Δ*mig1 *datasets in the case of the TF network (Additional file 7). Reporter features in case of *S1 *imply that the average score of neighbors of a particular feature is significantly higher than the average scores of randomly selected groups of genes (of the same size). This scoring system does not take into account the variability in the scores of neighbors. This may potentially lead to false positives in cases where only few high scoring neighbors (outliers) dominate the Reporter score. One way to overcome this problem is to use a scoring method where variance of node scores is accounted for. Towards this, we propose *S2 *and *S3 *where a high-scoring feature implies that the distribution of scores of the neighbors of the feature significantly differs from the distribution of scores for all of the nodes. Scores calculated by using *S2*, however, were observed to be well correlated to those calculated by using *S1 *(this can be attributed to Central Limit Theorem, due to which the t-distribution approaches normality in case of large number of data points.). Both *S1 *and *S2 *may suffer in case of extreme outliers as they will bias the mean and standard deviation. Scoring *S3 *is more suitable in such cases, since it is expected to be insensitive to the absolute magnitudes of scores. Finally, high-scoring features under *S4 *mean that the feature's neighbors have significant over-representation in nodes that pass a certain user-defined threshold score. This scoring feature is useful to evaluate whether a Reporter Feature is enriched in significantly changed genes, and can also be used to detect features with outlier nodes.

Scoring systems *S2*, *S3 *and *S4 *are not suitable for small groups and, thus, we suggest that they should not be applied to features with few neighbors (<3 for *S2 *and <5 for *S3 *and *S4*). Hyper-geometric score *S4 *additionally suffers from the requirement that a significance threshold needs to be set *a priori *in order to decide whether a node score is significant or not. Overall, we suggest that *S1 *is used primarily while *S3 *and *S4 *scores are manually inspected to avoid false positive *S1 *scores. The final interpretation of any of the Reporter Feature scores also necessarily depends on the way *p*-values are estimated for the genes in the network. Although most obvious application is for the *p*-values calculated across two different experimental conditions or mutants (e.g., by using *t*-test or U-test), it is also possible to use the algorithm with *p*-values (or scores) derived from other statistical tests (e.g. ANOVA). In such cases, the interpretation of Reporter Features must be rephrased accordingly.

### Reporter Features *versus *gene-set enrichment methods

A number of so-called gene-set enrichment methods reported in literature also offer the possibility of analyzing gene expression data in a biologically constrained way. Methods such as SGD GO Term Finder [28], BiNGO [29], MappFinder [30] and Onto-Express [31] use hypergeometric or binomial tests to evaluate over- and under-significance of representation of GO categories in a user-defined set of genes. Another popular (and computationally intensive) enrichment analysis method is the Gene Set Enrichment Analysis (GSEA) [32], which uses the gene expression of all transcripts in an array in order to score each gene-set for its enrichment in significantly changing genes. In particular, GSEA scores the enrichment of the gene group towards top (or bottom) of the overall list of genes ranked in order of decreasing significance.

In the Reporter Features algorithm, the gene-set is defined based on adjacency to a particular class of features, and the statistical test evaluates whether genes adjacent to these features are co-regulated. This notably differs from the above mentioned enrichment methods, where gene-sets are defined based on user criteria (often not based on biological adjacency), and the statistical test evaluates the enrichment of these sets in certain categories. In Supplementary Discussion (Additional file 8) we compare the results for the analysis of Δ*mig1 *using Reporter TF *vs *GSEA, and Reporter GO *vs *BiNGO, and discuss the biological meaning of the results. BiNGO and Reporter GO results overlap to some extent, but BiNGO required the *a priori *definition of a set of significantly changing genes, which is, *per se*, a very subjective process. Regarding the comparison with GSEA, we find Reporter Feature results biologically more meaningful and easier to interpret than GSEA results. Although information on the level of enrichment is interesting, being a method merely based on rank tests, GSEA misses sensitivity at the level of individual differential expression *p*-values. Additionally, while the score resulting from the Reporter Features algorithm gives a measure of co-regulation among all the neighbours of a feature, GSEA score simply hints at those features more enriched in a set of co-changed genes in a given experiment. We also note that Reporter GSEA was computationally much more demanding as opposed to Reporter Features calculation (Additional file 8).

### Limitations

Since the described method uses known biological information as underlying network structure for data analysis, the reliability of the included interaction data is not questioned by the algorithm. To our knowledge, this is also the case of other presently available methods that use network topology as data integration scaffold. We, however, note that it will be possible to include such reliability information, if available, by appropriately modifying the feature scoring system. Another limitation of our approach is that new potential feature-gene interactions can not be directly inferred from the analysis, but only *via *the intermediate features, as is the case for all network guided methods where only known interactions are used for data integration. Thus, methods such as clustering and promoter-sequence motif analysis [33] for a set of significantly changed genes/clusters are more suitable for such purposes. Moreover, for reconstructing the regulatory pathways, there are several data-driven tools available that will enable capturing new regulatory interactions [34,35]. Our study also does not address the dynamics of the bio-molecular interactions, which is an important feature of many cellular networks [20,36]. In such cases, methods that integrate dynamic features of the bio-molecules with the network topology, such as statistical analysis of network dynamics (SANDY) [36], are powerful for unraveling the underlying biological operational principles. Finally, we would also like to mention that Reporter Features identify "local" hot-spots in a network. To fully exploit the connectivity information it will be necessary to use global search algorithms such as sub-network finding [4,5]. Results from such global search methods, however, are more difficult to interpret biologically as they span multi-dimensional space with respect to biological features. Higher-degree Reporters provide a trade-off in this regard and enable limiting the feature-dimensionality of the results to a desired degree.

## Conclusion

The use of different biological networks to determine Reporter Features brings insights at different levels, ranging from global functional characterization to specific mechanistic aspects of cellular regulation (Figure 2). Although many genes overlap across these networks (Tables S13 and S14 in Additional file 8), the biological information gained is different due to the different connectivities in the different networks. This is due to the fact that the number of connections in a biological network is often far from the number of all possible connections (i.e. all-to-all interactions).

We applied Reporter Features for yeast glucose repression and human diabetes datasets, and this provided valuable information at three different biological dimensions, viz., protein interactions, functional families and transcriptional regulatory circuits. Both human diabetes and glucose repression phenomena were found to be related with global transcriptional responses affecting respiration and other cellular processes involved in energy generation. This regulatory architecture is uncovered by using an integrative and hypothesis-driven bottom-up approach, without *a priori *assumption regarding involvement of these processes.

Although similar scoring frameworks have been proposed for analyzing certain biological gene groups [30-32,37,38], these methods tackle the problem in a more data-driven fashion. Due to the hypothesis-driven nature of our proposed algorithm, based on a strong biological foundation, it is possible to systematically integrate multi-omics data in a multi-hypotheses fashion, thereby allowing us to discover fundamental and general modularity principles underlying the operation of biological systems. In particular, Reporter Features algorithm views the 'data + network' as a set of hypotheses pertaining to the biological information attributed to the edges in the network (e.g. an edge in a protein-DNA interaction network implies transcriptional regulation, while an edge in a protein-protein interaction network may imply signal transduction).

Our results provide evidence that the underlying hypothesis of Reporter Features algorithm (i.e., the cellular response to a perturbation is guided by the topology of bio-molecular interaction networks) is true to a large extent for several biological networks and, consequently, the cellular response to a perturbation can be modularized and characterized by using network topology information. This fundamental design rule for the transcriptional regulation can thus be used to identify hot-spots of regulation and gain information on the biological role of a particular genetic/environmental factor in an automated fashion without much *a priori *manual input of knowledge in a case-dependent fashion. Consequently, Reporter Features have the potential to be used as bio-markers and may also become a common tool for aiding in automated functional annotation of unknown or poorly characterized gene products.

## Methods

### Preparation of gene expression datasets

For both yeast and human transcriptome datasets, we used the CEL files supplied by the authors of the corresponding studies. Each dataset was normalized for intensities using dChip 1.3, and index expression calculation was also performed in dChip 1.3 using the PM-only model. In each dataset, only probesets with a Present call for all arrays were considered. All pair-wise statistical tests were performed using a 2-tail, heteroscedastic, Student's *t*-test.

### Selected biological networks

For the yeast *S. cerevisiae *we used the following sources of biological networks: Gene Ontology annotation from the *Saccharomyces *Genome Database [39] (SGD, Version: Revision 1.1199, 28/Oct/2005); information on regulators (transcription factors and regulatory proteins that directly or indirectly affect the expression of regulated genes) from the Yeast Proteome Database [14,40] (YPD, as of Mar/2007) and protein interactions from the Database of Interacting Proteins" [41] (DIP, as of 4/Dec/2005). The Gene Ontology annotation for *Homo sapiens *was obtained from GO Annotation @ European Bioinformatics Institute [26] (EBI, version 36.0, 21/Nov/2005).

The GO interaction graph was constructed connecting each GO term to all gene products annotated in that term (GO annotation as available from the GO gene-association file). The regulators interaction graph was constructed connecting each regulator to all genes known to be regulated by it. All network files used are available as SIF files in Additional file 9, Additional file 10, Additional file 11, Additional file 12 and Additional file 13.

### Inferred regulatory map for yeast Reporter TFs

In order to construct the inferred regulatory map for glucose repression using Reporter TFs information we selected for each mutant all the TFs with a *p*-value < 0.01 (i.e., threshold to be considered as Reporter TF). The graph was constructed connecting each Reporter TF to the corresponding node, i.e., to the component (gene) that was deleted. In Figure 3 we only represent TFs that were Reporter TFs for at least two of the perturbations.

## Availability and requirements

A flexible implementation of the Reporter Features algorithm described in this paper is available online. Project name: Reporter Features; Project home page: ; Operating system: Windows; Programming language: C++; Other requirements: none; Licence: free (no fees or software transfer agreements required) for academic, non-profit use. License available for commercial use upon request.

## Abbreviations

GO – Gene Ontology

TF – Transcription Factor

## Authors' contributions

APO designed the study, carried out most aspects of the work and drafted the manuscript. KRP designed the study, contributed with the new scoring systems, implemented the algorithm as an executable program and helped to draft the manuscript. JN conceived the study and participated in its design and coordination. All authors read and approved the final manuscript.

## Supplementary Material

Additional file 1 Supplementary Data 1 contains all sheets with feature scores mentioned in the text for yeast datasets, determined using scoring system *S1*. Click here for file

Additional file 2 Supplementary Table 1 contains the top-10 Reporter Gene Ontologies for the glucose repression knockout mutants, in the yeast case study. Click here for file

Additional file 3 Supplementary Table 2 contains the top-10 Reporter Proteins (first and second degree) for the Δ*grr1 *mutant, in the yeast case study. Click here for file

Additional file 4 Supplementary Table 3 contains the top-10 Reporter Complexes for the glucose repression knockout mutants, in the yeast case study. Click here for file

Additional file 5 Supplementary Data 2 contains all feature scores mentioned in the text for human diabetes datasets, determined using scoring system *S1*. Click here for file

Additional file 6 Supplementary Table 4 contains the top-10 Reporter Gene Ontologies for the human diabetes case study. Click here for file

Additional file 7 Supplementary Data 3 contains Reporter TF analysis for Δ*mig1 *and Δ*grr1 *using all scoring systems described in the main text (*S1 *to *S4*). Click here for file

Additional file 8 Supplementary Discussion contains comparisons of results using Reporter Features and using gene-set enrichment methods (namely, GSEA and BiNGO), and an overlap analysis between the different biological networks used in this study. Contains Supplementary Tables S10, S11, S12, S13 and S14. Click here for file

Additional file 9 Supplementary Table 5 contains the GO ontology annotation network for yeast. The file is supplied as TXT, but in SIF format (for example, it can be simply renamed .sif for usage under Cytoscape). Click here for file

Additional file 10 Supplementary Table 6 contains the TF regulatory network for yeast. The file is supplied as TXT, but in SIF format (for example, it can be simply renamed .sif for usage under Cytoscape). Click here for file

Additional file 11 Supplementary Table 7 contains the protein-protein interaction network for yeast. The file is supplied as TXT, but in SIF format (for example, it can be simply renamed .sif for usage under Cytoscape). Click here for file

Additional file 12 Supplementary Table 8 contains the MIPS Complexes association network for yeast. The file is supplied as TXT, but in SIF format (for example, it can be simply renamed .sif for usage under Cytoscape). Click here for file

Additional file 13 Supplementary Table 9 contains the GO ontology annotation network for human. The file is supplied as TXT, but in SIF format (for example, it can be simply renamed .sif for usage under Cytoscape). Click here for file
