# Supplementary material for: Architecture of transcriptional regulatory circuits is knitted over the topology of bio-molecular interaction networks
Source: BMC Syst Biol. 2008 Feb 8;2:17. doi: 10.1186/1752-0509-2-17 (PMC2268660; doi:10.1186/1752-0509-2-17)
Supplement: Additional file 2 — Supplementary Table 1 contains the top-10 Reporter Gene Ontologies for the glucose repression knockout mutants, in the yeast case study. [file 1752-0509-2-17-S2.doc]

**Supplementary Table 1** - Top-10 Reporter Gene Ontologies for the glucose repression knockout mutants, in the yeast case study. Gene Ontologies are ranked by their *Z*-score and *N* is the number of gene-nodes annotated within each category. GO categories include biological processes (P), molecular functions (F) and cellular compartments (C). (see Supplementary data 1 for the complete list)

| **RS vs** **Δ*GRR1*** | | | **RS vs Δ*HXK2*** | | | **RS vs Δ*MIG1MIG2*** | | | **RS vs Δ*MIG1*** | | |
| --- | --- | --- | --- | --- | --- | --- | --- | --- | --- | --- | --- |
| **GO category (P/F/C)** | **Z** | **N** | **GO category (P/F/C)** | **Z** | **N** | **GO category (P/F/C)** | **Z** | **N** | **GO category (P/F/C)** | **Z** | **N** |
| ATP synthesis coupled proton transport (P) | 8.06 | 16 | aerobic respiration (P) | 7.93 | 63 | ATP synthesis coupled proton transport (P) | 7.97 | 16 | fructose transporter activity (F) | 3.39 | 10 |
| aerobic respiration (P) | 7.66 | 63 | mitochondrial large ribosomal subunit (C) | 7.70 | 43 | mitochondrial large ribosomal subunit (C) | 6.94 | 43 | hexose transport (P) | 3.39 | 10 |
| hydrogen-transporting ATP synthase activity, rotational mechanism (F) | 6.54 | 11 | ATP synthesis coupled proton transport (P) | 7.16 | 16 | mitochondrial small ribosomal subunit (C) | 6.75 | 33 | mannose transporter activity (F) | 3.39 | 10 |
| mitochondrial large ribosomal subunit (C) | 6.52 | 43 | tricarboxylic acid cycle (P) | 6.47 | 15 | hydrogen-transporting ATP synthase activity, rotational mechanism (F) | 6.50 | 11 | nucleolus (C) | 2.97 | 148 |
| mitochondrion (C) | 6.08 | 590 | mitochondrion (C) | 6.44 | 590 | aerobic respiration (P) | 6.11 | 63 | molecular function unknown (F) | 2.96 | 1742 |
| tricarboxylic acid cycle (P) | 5.89 | 15 | mitochondrial small ribosomal subunit (C) | 6.28 | 33 | protein biosynthesis (P) | 4.84 | 245 | translation initiation factor activity (F) | 2.74 | 28 |
| mitochondrial small ribosomal subunit (C) | 5.25 | 33 | hydrogen-transporting ATP synthase activity, rotational mechanism (F) | 6.26 | 11 | proton-transporting ATP synthase complex, coupling factor F(o) (C) | 4.60 | 5 | glycerol catabolism (P) | 2.73 | 3 |
| proton-transporting ATP synthase complex, coupling factor F(o) (C) | 4.69 | 5 | mitochondrial inner membrane (C) | 6.23 | 98 | structural constituent of ribosome (F) | 4.42 | 199 | glucose transporter activity (F) | 2.68 | 13 |
| cytochrome-c oxidase activity (F) | 4.43 | 9 | cytochrome-c oxidase activity (F) | 4.83 | 9 | cytochrome-c oxidase activity (F) | 4.32 | 9 | glycerone kinase activity (F) | 2.60 | 2 |
| respiratory chain complex IV (C) | 4.43 | 9 | respiratory chain complex IV (C) | 4.83 | 9 | respiratory chain complex IV (C) | 4.32 | 9 | asparagine-tRNA ligase activity (F) | 2.59 | 2 |
