# Supplementary material for: Architecture of transcriptional regulatory circuits is knitted over the topology of bio-molecular interaction networks
Source: BMC Syst Biol. 2008 Feb 8;2:17. doi: 10.1186/1752-0509-2-17 (PMC2268660; doi:10.1186/1752-0509-2-17)
Supplement: Additional file 3 — Supplementary Table 2 contains the top-10 Reporter Proteins (first and second degree) for the Δgrr1 mutant, in the yeast case study. [file 1752-0509-2-17-S3.doc]

**Supplementary Table 2** - Top-10 Reporter Proteins (first and second degree) for the *Δgrr1* mutant, in the yeast case study. Proteins are ranked by their *Z*-score. *N* is the number of neighbors. (see Supplementary data 1 for complete list)

| RS vs **Δ***GRR1* | | | RS vs **Δ***GRR1* | | |
| --- | --- | --- | --- | --- | --- |
| Protein (1st-degree) | Z | N | Protein (2nd-degree) | Z | N |
| ATP1 | 7.44 | 16 | TIM11 | 7.39 | 20 |
| ATP2 | 7.20 | 19 | ATP16 | 7.06 | 19 |
| ATP6 | 5.12 | 7 | ATP7 | 6.77 | 43 |
| CDC14 | 3.92 | 27 | ATP5 | 6.72 | 43 |
| ATP7 | 4.11 | 7 | ATP18 | 6.38 | 25 |
| ATP18 | 4.11 | 6 | ATP6 | 6.16 | 24 |
| ATP17 | 3.73 | 6 | ATP17 | 5.78 | 24 |
| MRP4 | 3.58 | 35 | ATP11 | 4.09 | 35 |
| FES1 | 3.37 | 11 | ATP4 | 3.72 | 108 |
| MRPL7 | 3.25 | 4 | IMG1 | 3.70 | 58 |
