# Supplementary material for: Architecture of transcriptional regulatory circuits is knitted over the topology of bio-molecular interaction networks
Source: BMC Syst Biol. 2008 Feb 8;2:17. doi: 10.1186/1752-0509-2-17 (PMC2268660; doi:10.1186/1752-0509-2-17)
Supplement: Additional file 4 — Supplementary Table 3 contains the top-10 Reporter Complexes for the glucose repression knockout mutants, in the yeast case study. [file 1752-0509-2-17-S4.doc]

**Supplementary Table 3** - Top-10 Reporter Complexes for the glucose repression knockout mutants, in the yeast case study. MIPS complexes are ranked by their *Z*-score and *N* is the number of gene-nodes annotated within each category. (see Supplementary data 1 for the complete list)

| **RS vs** **Δ*GRR1*** | | | **RS vs Δ*HXK2*** | | | **RS vs Δ*MIG1MIG2*** | | | **RS vs Δ*MIG1*** | | |
| --- | --- | --- | --- | --- | --- | --- | --- | --- | --- | --- | --- |
| **MIPS Complex** | **Z** | **N** | **MIPS Complex** | **Z** | **N** | **MIPS Complex** | **Z** | **N** | **MIPS Complex** | **Z** | **N** |
| F0/F1 ATP synthase (complex V) | 7.23 | 15 | Mitochondrial ribosomal large subunit | 7.85 | 41 | F0/F1 ATP synthase (complex V) | 7.79 | 15 | Complex Number 321 (Ho Y, et al.)  (*protein biosynthesis) | 3.52 | 6 |
| Mitochondrial ribosomal large subunit | 6.69 | 41 | F0/F1 ATP synthase (complex V) | 6.46 | 15 | Mitochondrial ribosomal large subunit | 6.85 | 41 | Complex Number 380 (Ho Y, et al.)  (*translation initiation factors) | 2.88 | 13 |
| Mitochondrial ribosomal small subunit | 5.03 | 29 | Mitochondrial ribosomal small subunit | 6.08 | 29 | Mitochondrial ribosomal small subunit | 6.76 | 29 | Complex Number 88 (Ho Y, et al.)  (*carbohydrate metabolism) | 2.76 | 11 |
| Cytochrome c oxidase (complex IV) | 4.73 | 8 | Cytochrome c oxidase (complex IV) | 5.05 | 8 | Other respiration chain complexes | 4.62 | 14 | Tim22p-complex | 2.65 | 5 |
| Other respiration chain complexes | 3.96 | 14 | Complex Number 108, probably protein synthesis turnover (Gavin AC, et al.) | 4.81 | 52 | Complex Number 104, probably protein synthesis turnover (Gavin AC, et al.) | 4.30 | 39 | Complex Number 70 (Ho Y, et al.)  (COQ7, COR1, IME4, PRP28, YJL068C) | 2.59 | 5 |
| Cytochrome bc1complex (Ubiquinol-cytochrome c reductase complex, complexIII) | 3.90 | 9 | Other respiration chain complexes | 4.77 | 14 | Cytochrome c oxidase (complex IV) | 4.14 | 8 | Complex Number 309 (Ho Y, et al.)  (RIM15, PHO85) | 2.48 | 2 |
| Complex Number 47, probably intermediate and energy metabolism (Gavin AC, et al.) | 3.85 | 4 | Complex Number 104, probably protein synthesis turnover (Gavin AC, et al.) | 4.68 | 39 | 2-oxoglutarate dehydrogenase | 3.70 | 3 | Complex Number 51, eIF3 (Krogan NJ, et al.) | 2.48 | 9 |
| Succinate dehydrogenase complex (complex II) | 4.05 | 4 | Succinate dehydrogenase complex (complex II) | 4.00 | 4 | Cytochrome bc1 complex (Ubiquinol-cytochrome c reductase complex, complex III) | 3.62 | 9 | Complex Number 210, probably transcription/ DNA maintanance/ chromatin structure (Gavin AC, et al.) | 2.42 | 18 |
| Complex Number 37 (Ho Y,et al.) | 4.01 | 17 | Mitochondrial splicing complexes | 3.91 | 12 | Mitochondrial splicing complexes | 3.41 | 12 | Complex Number 96 (Ho Y, et al.)  (*nucleolar and ribosome biogenesis) | 2.39 | 16 |
| Complex Number 104, probably protein synthesis turnover (Gavin AC, et al.) | 3.64 | 39 | Complex Number 47, probably intermediate and energy metabolism (Gavin AC, et al.) | 3.85 | 4 | Complex Number 108, probably protein synthesis turnover (Gavin AC, et al.) | 3.38 | 52 | Complex Number 87, probably protein synthesis turnover (Gavin AC, et al.) | 2.29 | 6 |
