# Supplementary material for: Architecture of transcriptional regulatory circuits is knitted over the topology of bio-molecular interaction networks
Source: BMC Syst Biol. 2008 Feb 8;2:17. doi: 10.1186/1752-0509-2-17 (PMC2268660; doi:10.1186/1752-0509-2-17)
Supplement: Additional file 6 — Supplementary Table 4 contains the top-10 Reporter Gene Ontologies for the human diabetes case study. [file 1752-0509-2-17-S6.doc]

**Supplementary Table 4** - Top-10 Reporter Gene Ontologies for the human diabetes case study. Gene Ontologies are ranked by their *Z*-score and *N* is the number of gene-nodes annotated within each category. GO categories include biological processes (P), molecular functions (F) and cellular compartments (C). (see Supplementary data 2 for complete list)

| **DM vs FH-** | | | **DM vs FH+** | | | **FH- vs FH+** | | |
| --- | --- | --- | --- | --- | --- | --- | --- | --- |
| **GO category (P/F/C)** | **Z** | **N** | **GO category (P/F/C)** | **Z** | **N** | **GO category (P/F/C)** | **Z** | **N** |
| protein biosynthesis (P) | 8.78 | 136 | protein biosynthesis (P) | 8.91 | 136 | protein biosynthesis (P) | 7.39 | 136 |
| structural constituent of ribosome (F) | 7.25 | 84 | RNA binding (F) | 7.93 | 207 | RNA binding (F) | 6.91 | 207 |
| mitochondrion (C) | 6.86 | 228 | structural constituent of ribosome (F) | 6.83 | 84 | mitochondrion (C) | 6.65 | 228 |
| RNA binding (F) | 6.85 | 207 | mitochondrion | 6.67 | 228 | structural constituent of ribosome (F) | 5.48 | 84 |
| ribosome (C) | 6.72 | 70 | ribosome | 6.10 | 70 | ubiquitin cycle (P) | 5.26 | 62 |
| ubiquitin cycle (P) | 5.79 | 62 | ubiquitin-dependent protein catabolism (P) | 5.64 | 43 | ribosome (C) | 5.24 | 70 |
| ubiquitin-dependent protein catabolism (P) | 5.55 | 43 | muscle development (P) | 5.27 | 83 | ubiquitin-dependent protein catabolism (P) | 5.08 | 43 |
| muscle development (P) | 5.39 | 83 | ubiquitin cycle (P) | 5.10 | 62 | hydrogen-transporting ATPase activity, rotational mechanism (F) | 4.75 | 21 |
| protein folding (P) | 4.42 | 86 | translation elongation factor activity (F) | 4.92 | 10 | ATP synthesis coupled proton transport (P) | 4.75 | 21 |
| DNA-directed RNA polymerase activity (F) | 3.93 | 15 | protein folding (P) | 4.70 | 86 | muscle development (P) | 4.68 | 83 |
