# Supplementary material for: Architecture of transcriptional regulatory circuits is knitted over the topology of bio-molecular interaction networks
Source: BMC Syst Biol. 2008 Feb 8;2:17. doi: 10.1186/1752-0509-2-17 (PMC2268660; doi:10.1186/1752-0509-2-17)
Supplement: Additional file 8 — Supplementary Discussion contains comparisons of results using Reporter Features and using gene-set enrichment methods (namely, GSEA and BiNGO), and an overlap analysis between the different biological networks used in this study. Contains Supplementary Tables S10, S11, S12, S13 and S14. [file 1752-0509-2-17-S8.doc]

**Supplementary Discussion**

**Architecture of transcriptional regulatory circuits is knitted over the topology of bio-molecular interaction networks**

Ana Paula Oliveira, Kiran Raosaheb Patil, Jens Nielsen

-

# Reporter TF *versus* GSEA

We run both the Reporter Features algorithm and the GSEA (Gene Set Enrichment Analysis) [1] using the gene expression dataset for *Δmig1* and the TF regulatory network (Supplementary Table 6). For Reporter Features, we use the ‘distribution of means’ scoring system. The 23 Reporter TFs (*p*-value < 0.05) are listed in Table S10, ranked in decreasing order of significance. GSEA was run using the Java desktop application available at http://www.broad.mit.edu/gsea/software/software_index.html. In order to use GSEA using exactly the same data, the regulatory network was converted from SIF format into GMT format. In javaGSEA, the following changes were made to the default parameters: collapse – *false*; metric – *tTest*; set_min – *2*. Applying GSEA to *Δmig1* dataset yielded no significant gene-sets at a false discovery rate <25%. Since GSEA nominal *p*-values are not normalized to group size, we decided to take the top 23 gene-sets ranked based on their nominal enrichment score (NES) in order to compare them with our Reporter TFs. The results from GSEA are presented in Table S11. In terms of computational time, it took 1 minute to calculate Reporter TFs and 20 minutes to compute the GSEA output using a Intel Centrino machine running at 1500 MHz and with 1.0 GB of RAM.

For the case under study, we observed that Reporter TFs better correspond with the biologically expected results than those obtained with GSEA. Although this “expectancy” is somehow subjective and limited to the existing knowledge, we consider it to be fundamental for validation. Having remarked this, we observe that Mig1 comes at the top of the Reporter TF list, which is meaningful since genes under the regulation of Mig1 are those expected to be transcriptionally more affected when Mig1 is not present. Other regulators (e.g., Mig2, Grr1, Mth1, Snf1, Snf3, Rgt2, Gal4) known to be affected in the glucose repression signal transduction pathways also show up in the Reporters list. On the other side, with GSEA no gene-sets were significant at a false discovery rate less than 25%. Nevertheless, if we consider the top scoring gene-sets (based on NES), Mig1 also shows up, although in the middle of the list. From the top-5 gene-sets, only one is related with glucose repression (Ngg1), while the others are involved in chromatin remodelling (Snf6), histone acetylation (Eaf1), protein folding (Ssa1) and transcriptional regulation (Mot1). Overall, there are only 5 regulators in common between Tables S10 and S11 (Mig1, Mig2, Spt6, Snf6 and Gal4).

| Table S10 – Reporter TF (p-value <0.05) for the *Δmig1* dataset, using the regulatory network of Supplementary Table 6. | Table S11 – GSEA top-scoring gene-sets for the *Δmig1* dataset, and using the regulatory network of Supplementary Table 6. |
| --- | --- |
| | **TF** | **N** | **Z-score** | **p-value** | | --- | --- | --- | --- | | MIG1 | 22 | 2.65105 | 0.004012 | | MIG2 | 11 | 2.63998 | 0.004146 | | GRR1 | 6 | 2.48649 | 0.00645 | | MTH1 | 4 | 2.37838 | 0.008694 | | ARG82 | 8 | 2.27053 | 0.011588 | | DST1 | 3 | 2.23008 | 0.012871 | | SPT16 | 13 | 2.18019 | 0.014622 | | MOT3 | 17 | 2.14557 | 0.015954 | | STP1 | 2 | 2.11104 | 0.017385 | | SPT6 | 4 | 2.10996 | 0.017431 | | SNF6 | 2 | 2.10143 | 0.017801 | | KCS1 | 2 | 2.04624 | 0.020366 | | GAL4 | 12 | 2.00256 | 0.022613 | | RAD26 | 6 | 1.96116 | 0.02493 | | SNF1 | 23 | 1.94333 | 0.025988 | | SNF2 | 23 | 1.90159 | 0.028613 | | SWI1 | 16 | 1.87594 | 0.030332 | | RFA2 | 9 | 1.87515 | 0.030386 | | GZF3 | 4 | 1.86134 | 0.031348 | | HTZ1 | 2 | 1.81605 | 0.034682 | | RGT2 | 5 | 1.71833 | 0.042869 | | CDC42 | 3 | 1.6728 | 0.047183 | | SNF3 | 3 | 1.65259 | 0.049207 | | | **Name of Gene-set** | **Size** | **ES** | **NES** | **FWER  p-value** | | --- | --- | --- | --- | --- | | NGG1 | 4 | 0.822053 | 1.595041 | 0.493 | | SNF6 | 2 | 0.739847 | 1.517796 | 0.748 | | EAF3 | 3 | 0.776264 | 1.501656 | 0.748 | | SSA1 | 2 | 0.839301 | 1.496463 | 0.748 | | MOT2 | 4 | 0.566847 | 1.488036 | 0.748 | | SOK2 | 2 | 0.91497 | 1.47748 | 0.748 | | IWS1 | 8 | 0.531246 | 1.447451 | 0.856 | | GAL4 | 12 | 0.550351 | 1.431722 | 0.913 | | OPI1 | 13 | 0.472744 | 1.401535 | 0.958 | | SWI3 | 4 | 0.624341 | 1.394017 | 1 | | ESA1 | 4 | 0.785075 | 1.375317 | 1 | | MIG1 | 22 | 0.524495 | 1.363566 | 1 | | SPT6 | 4 | 0.565298 | 1.358004 | 1 | | SPT10 | 5 | 0.566519 | 1.354018 | 1 | | SPT21 | 5 | 0.566519 | 1.354018 | 1 | | MIG2 | 11 | 0.546989 | 1.337885 | 1 | | REG1 | 4 | 0.543746 | 1.334582 | 1 | | SPT7 | 6 | 0.554221 | 1.323323 | 1 | | GAS1 | 6 | 0.635954 | 1.301921 | 1 | | CIN5 | 3 | 0.864664 | 1.301137 | 1 | | HXT1 | 2 | 0.796286 | 1.297391 | 1 | | SNF5 | 7 | 0.466154 | 1.283838 | 1 | | VPS24 | 2 | 0.893501 | 1.265127 | 1 | |

Having used the scoring system (s1), ‘distribution of means’, to calculate Reporters, a high-score Reporter TF means that the neighbours of that TF (i.e., the genes know to be regulated by the TF) are, as a group, changing their transcriptional response significantly more than any random ‘same-size’ group of genes picked from the whole array. Since the algorithm normalizes both for background and for the size of the network, it is valid to compare the z-score (or *p*-values) of Mig1 (22 neighbours), Mig2 (11 neighbours) and Grr1 (6 neighbours) and conclude that the deletion of Mig1 in the mutant *Δmig1*, when comparing with the wild-type, mostly affects the transcription of the neighbours of Mig1, followed by the neighbours of Mig2 and the neighbours of Grr1.

In the case of GSEA, a high normalized enrichment score (NES) means that the gene-set (i.e., the neighbours of the TF) is highly enriched in genes that rank high in the list of *p*-values ranked in order of increasing probability (*p*-values from a t-test on the differential expression of *Δmig1* and wild-type). For example, in the case under study, 8 of the 22 neighbours of Mig1 define the enrichment score of the ‘Mig1 group’, with 3 of the genes belonging to the top-30 of the ranked list of p-values. Although information on the level of enrichment is interesting, being a method merely based on rank tests, GSEA misses sensitivity at the level of differential expression. Additionally, while the score resulting from the Reporter Features algorithm gives a measure of co-regulation among all the neighbours of a feature, GSEA score only hints at those features more enriched in co-regulated genes.

# Reporter GO *versus* BiNGO

Since enrichment methods are commonly available for analysis of over-representation of GO terms, we compared the outputs of BiNGO [2] and Reporter GO. We also run GSEA using the same network as for Reporters, but this turn out to be computationally too intense, and therefore we did not managed to include GSEA in this comparison. In this example we use the gene expression dataset for *Δmig1*. For Reporter GOs, we use the GO term network (Supplementary Table 5) and the ‘distribution of means’ scoring system. The 114 Reporter GOs (*p*-value < 0.05) are listed in Table S12, ranked in decreasing order of significance. BiNGO was run using the Cytoscape plug-in. We previously selected the genes from the *Δmig1 vs* wild-type dataset that changed their expression level at a significance level *p*-value<0.05 (354 genes). On this group, we used BiNGO to perform a hypergeomtric test for over-representation, and got as output 71 GO categories significant at *p*-value < 0.05 (without correction). BiNGO results are also displayed in Table S12.

Although overall results for both Reporter GO and BiNGO refer to overlapping processes/functions/components of the cell, interpretation of the results obtained with the two methods is different. For BiNGO, one needed to select *a priori* a set of significant changing genes, and results tell how much the different GO categories are enriched in these significant changing genes. On the other side, Reporter GOs outputs GO terms whose neighbours genes are, as a group, changing their response more significantly than random networks of the same size, which can be seen as a measure of significant overall co-regulation. From Table S12 we can observe that some of the GO terms only show up in the Reporter analysis – namely the GO terms “molecular function unknown”, “biological processes unknown” and “cellular components unknown”. Despite that these are very general terms, the fact that they appear as Reporter GOs clearly indicates that many of the genes changing their expression in response to *MIG1* deletion still have unidentified function, and this cannot be captured by an enrichment method, since these categories are too large to become significant in an enrichment test.

Table S12 – Reporter GOs (p-value <0.05) and over-represented genes using BiNGO (Full GO) for the *Δmig1* dataset. The GO terms are listed in decreading order of significance. In bold are common GO between the two methods.

| **Reporter GO** | **BiNGO (Full GO)** |
| --- | --- |
| **fructose transporter activity** | quinone cofactor biosynthesis |
| **hexose transport** | **ubiquinone metabolism** |
| **mannose transporter activity** | ubiquinone biosynthesis |
| nucleolus | quinone cofactor metabolism |
| molecular function unknown | **glycerone kinase activity** |
| translation initiation factor activity | **aromatic-amino-acid transaminase activity** |
| **glycerol catabolism** | **asparagine-tRNA ligase activity** |
| **glucose transporter activity** | **plasma membrane** |
| **glycerone kinase activity** | coenzyme biosynthesis |
| **asparagine-tRNA ligase activity** | GTPase regulator activity |
| **ubiquinone metabolism** | inner mitochondrial membrane organization and biogenesis |
| guanine nucleotide transport | intramolecular oxidoreductase activity, interconverting aldoses and ketoses |
| guanine nucleotide transporter activity | cofactor biosynthesis |
| translational initiation | mitochondrial membrane organization and biogenesis |
| **aromatic-amino-acid transaminase activity** | **regulation of exit from mitosis** |
| aromatic amino acid family metabolism | **GTPase activator activity** |
| basic amino acid transport | **regulation of mitotic recombination** |
| **proline biosynthesis** | **proline biosynthesis** |
| glucose transport | **glycerol catabolism** |
| RNA elongation from RNA polymerase II promoter | polyol catabolism |
| **GTPase activator activity** | **fructose transporter activity** |
| glutamate 5-kinase activity | **mannose transporter activity** |
| transcription factor TFIID complex | **hexose transport** |
| MAPKKK cascade during osmolarity sensing | monosaccharide transport |
| lipid raft | **glucose transporter activity** |
| mitotic chromosome condensation | isomerase activity |
| **regulation of mitotic recombination** | processing of 20S pre-rRNA |
| protein deneddylation | symporter activity |
| microtubule cytoskeleton organization and biogenesis | hexose transporter activity |
| coproporphyrinogen oxidase activity | monosaccharide transporter activity |
| cellular component unknown | cofactor binding |
| vitamin transport | four-way junction DNA binding |
| **synaptonemal complex** | nucleoside catabolism |
| tRNA processing | **removal of nonhomologous ends** |
| phosphopantothenate-cysteine ligase activity | spindle orientation checkpoint |
| COPII-coated vesicle | mitochondrial inner membrane protein insertion complex |
| spindle pole | snoRNA binding |
| structural constituent of nuclear pore | coenzyme metabolism |
| protein import into nucleus, docking | amine transporter activity |
| H4/H2A histone acetyltransferase complex | metallopeptidase activity |
| DNA replication and chromosome cycle | mRNA cleavage factor complex |
| threonine synthase activity | metalloendopeptidase activity |
| mature ribosome assembly | telomere maintenance via telomerase |
| basic amino acid transporter activity | tRNA-pseudouridine synthase activity |
| di-, tri-valent inorganic cation transport | **synaptonemal complex** |
| water transport | extrinsic to mitochondrial inner membrane |
| water channel activity | DNA topoisomerase activity |
| DNA dealkylation | cofactor metabolism |
| biological process unknown | mitochondrial inner membrane |
| asparaginyl-tRNA aminoacylation | sugar transporter activity |
| motor activity | response to heat |
| phosphate transport | enzyme activator activity |
| dihydroorotase activity | amino acid transporter activity |
| pyrimidine nucleotide biosynthesis | condensed nuclear chromosome |
| protein import into mitochondrial inner membrane | regulation of DNA recombination |
| magnesium ion transport | structure-specific DNA binding |
| inorganic diphosphatase activity | organelle lumen |
| pentose-phosphate shunt | membrane-enclosed lumen |
| transcription termination from RNA polymerase II promoter | cytoplasm organization and biogenesis |
| methionine biosynthesis | ribosome biogenesis and assembly |
| ubiquitin-specific protease activity | isoprenoid biosynthesis |
| protein geranylgeranyltransferase activity | pseudouridine synthase activity |
| CAAX-protein geranylgeranyltransferase complex | transferase activity, transferring aldehyde or ketonic groups |
| membrane organization and biogenesis | isoprenoid metabolism |
| organic acid transport | carbohydrate transport |
| protein kinase regulator activity | ribosomal large subunit biogenesis |
| para-aminobenzoic acid metabolism | meiosis I |
| 4-amino-4-deoxychorismate synthase activity | transaminase activity |
| regulation of pH | transferase activity, transferring nitrogenous groups |
| aerobic respiration | extrinsic to plasma membrane |
| **regulation of exit from mitosis** | NADPH regeneration |
| farnesyltranstransferase activity |  |
| terpenoid biosynthesis |  |
| Rho GTPase activator activity |  |
| error-free DNA repair |  |
| 3'(2'),5'-bisphosphate nucleotidase activity |  |
| hyperosmotic salinity response |  |
| endoplasmic reticulum lumen |  |
| GDP-mannose biosynthesis |  |
| triplex DNA binding |  |
| MIND complex |  |
| bud site selection |  |
| propionate metabolism |  |
| vitamin transporter activity |  |
| negative regulation of transcription from RNA polymerase II promoter by pheromones |  |
| **plasma membrane** |  |
| trehalose-phosphatase activity |  |
| dephospho-CoA kinase activity |  |
| DRAP deaminase activity |  |
| snoRNA transcription |  |
| phosphoric ester hydrolase activity |  |
| thiamin diphosphokinase activity |  |
| thiamin diphosphate biosynthesis |  |
| inorganic phosphate transporter activity |  |
| glucan 1,3-beta-glucosidase activity |  |
| sphingolipid transporter activity |  |
| purine-nucleoside phosphorylase activity |  |
| purine nucleoside catabolism |  |
| ubiquitin ligase complex |  |
| sodium ion homeostasis |  |
| copper chaperone activity |  |
| FAD binding |  |
| spindle midzone |  |
| ion transporter activity |  |
| G1-specific transcription in mitotic cell cycle |  |
| low affinity iron ion transport |  |
| DNA topological change |  |
| anthranilate synthase activity |  |
| anthranilate synthase complex |  |
| multidrug transporter activity |  |
| **removal of nonhomologous ends** |  |
| APG12 activating enzyme activity |  |
| APG8 activating enzyme activity |  |
| alkylbase DNA N-glycosylase activity |  |

# Level of overlap between the different biological networks

The different networks used in this study (GO annotation network, TF regulatory network, protein-protein interaction network and complexes interaction) and in our previous study [3] (metabolic network) cover different aspects of cell function and regulation, as illustrated in Figure 2A of the main text. In order to analyze the level of overlap between genes belonging to the different networks, we prepared Tables S13 and S14. Despite the fact that different networks span different parts of the cell, there is a certain level of overlap that allows different levels of regulation to be detected by more than one Reporter Feature analysis.

Table S13 – Number of genes overlapping between two networks. In brackets are the total number of genes belonging to each of the networks.

| **Number of genes associated with:** | ***GO term (5723)*** | ***PPI (4616)*** | ***MIPS complex (2734)*** | ***TF (1052)*** | ***Metabolic Network (612)*** |
| --- | --- | --- | --- | --- | --- |
| ***GO term (5723)*** |  | 4238 | 2661 | 1029 | 608 |
| ***PPI (4616)*** | 4238 |  | 2439 | 795 | 474 |
| ***MIPS complex (2734)*** | 2661 | 2439 |  | 493 | 252 |
| ***TF (1052)*** | 1029 | 795 | 493 |  | 292 |
| ***Metabolic Network (612)*** | 608 | 474 | 252 | 292 |  |

Table S14 – Number of genes overlapping between three or more networks.

| GO & TF & MIPS complex | 489 |
| --- | --- |
| GO & TF & PPI | 780 |
| GO & TF & Metabolic | 291 |
| GO & MIPS complex & PPI | 2389 |
| GO & MIPS complex & Metabolic | 250 |
| GO & PPI & Metabolic | 472 |
| TF & MIPS complex & PPI | 433 |
| TF & MIPS complex & Metabolic | 131 |
| TF & PPI & Metabolic | 242 |
| MIPS complex & PPI & Metabolic | 238 |
| GO & TF & MIPS complex & PPI | 429 |
| GO & TF & MIPS complex & Metabolic | 140 |
| TF & MIPS complex & PPI & Metabolic | 135 |
| GO & TF & MIPS complex & PPI & Metabolic | 134 |

Reference List

1. Subramanian A, Tamayo P, Mootha VK, Mukherjee S, Ebert BL, Gillette MA, Paulovich A, Pomeroy SL, Golub TR, Lander ES et al.: **Gene set enrichment analysis: a knowledge-based approach for interpreting genome-wide expression profiles.** *Proc Natl Acad Sci U S A* 2005, **102:**15545-15550.

2. Maere S, Heymans K, Kuiper M: **BiNGO: a Cytoscape plugin to assess overrepresentation of gene ontology categories in biological networks.** *Bioinformatics* 2005, **21:**3448-3449.

3. Patil KR, Nielsen J: **Uncovering transcriptional regulation of metabolism by using metabolic network topology.** *Proc Natl Acad Sci U S A* 2005, **102:**2685-2689.
